# Characterization and phylogenetic analysis of the complete mitochondrial genome of *Eleutheronema rhadinum* (Perciforms: Polynemidae)

**DOI:** 10.1080/23802359.2021.2016081

**Published:** 2022-02-24

**Authors:** Lei Zeng, Pengfei Wang

**Affiliations:** Key Laboratory of South China Sea Fishery Resources Exploitation & Utilization, Ministry of Agriculture, South China Sea Fisheries Research Institute, Chinese Academy of Fishery Sciences, Guangzhou, PR China

**Keywords:** Mitochondrial genome, *Eleutheronema rhadinum*, Polynemidae, phylogenetic

## Abstract

This study aimed to elucidate the complete mitochondrial genome (mitogenome) of *Eleutheronema rhadinum* (Perciforms: Polynemidae). The circular mitogenome was 16,556 bp in size, with the base composition: A − 28.17%, T − 26.99%, C − 28.72%, and G − 16.12%. It had the typical vertebrate mitochondrial gene arrangement and was made up of 13 protein-coding genes, 22 tRNA genes, two ribosomal RNA genes, and one non-coding control region. Phylogenetic analysis using maximum-likelihood approved that *E. rhadinum* belonged to the family Polynemidae and had close relationship to *E. tetradactylum*. The present data will contribute to future phylogenetic studies on members of family Polynemidae and conservation strategies for *E. rhadinum*.

The East Asian fourfinger threadfin, *Eleutheronema rhadinum*, belongs to Eleutheronema, Polynemidae family. It is a commercially crucial species for coastal and inshore small-scale fisheries in East Asia (Motomura 2003; Su et al. 2020). Till date, there has been much disagreement concerning the relationships of Polynemidae family and taxonomic status (Li et al. 2016). Studies have showed that Eleutheronema of Polynemidae family consists of three valid species: *Eleutheronema tetradactylum*, *E. rhadinum*, and *E. tridactylum*. The mitochondrial genome of *E. tetradactylum* has been determined, while that of *E. rhadinum* and *E. tridactylum* are unknown yet. In this study, the complete mitochondrial genome of *E. rhadinum* was determined and described, and phylogenetic relationship of *E. rhadinum* with all mitogenome-determined Eleutheronema species and 13 representative Perciforms species was analyzed. We expect that the present result will provide useful molecular data for protection of the germplasm resources of *E. rhadinum* and contributing to further explore the phylogenetic evolution of species in Polynemidae.

The specimen was collected from the South China Sea (22° 026′N, 113° 271′E) in August 2019. The muscle sample was stored at −80 °C and deposited in South China Sea Fisheries Research Institute, Chinese Academy of Fishery Sciences (email: zenglei@scsfri.ac.cn) under the voucher number SCSFRI-20190809002. Whole-genome sequencing was performed by next-generation sequencing using the Illumina HiSeq 6000 Sequencing System (Illumina, Inc., San Diego, CA). Clean data were assessed and assembled by FastQC v0.11.4 (www.bioinformatics.babraham.ac.uk/bugzilla) and NOVOPlasty (https://github.com/ndierckx/NOVOPlasty. The tRNA genes were identified using tRNAscan-SE 2.0 (Lowe and Chan 2016). The rRNA and protein-coding genes (PCGs) were identified and confirmed via multiple sequence alignment with homologous genes from published mitochondrial genomes of other species in Polynemidae. Phylogenetic analysis was conducted by maximum-likelihood (ML) method using MEGA X software with 1000 bootstrap replication based on complete mitochondrial genomes of species in Perciforms (Kumar et al. 2018).

The complete mitogenome of *E. rhadinum* was 16,556-bp-long (GenBank accession number: MW630081). The overall base composition was 28.17% A, 26.99% T, 28.72% C, and 16.12% G, with a slight A + T bias of 55.16%. It had the typical vertebrate mitochondrial gene arrangement (Wang et al. 2018) and consisted of 13 PCGs, 22 tRNAs, two rRNAs, and one control region (CR). Most genes were encoded on the heavy strand, except *ND6* and eight tRNA genes (*tRNA-Gln*, *tRNA-Ala*, *tRNA-Asn*, *tRNA-Cys*, *tRNA-Tyr*, *tRNA-Ser*^(^*^GCU^*^)^, *tRNA-Glu*, and *tRNA-Pro*), which were encoded on the light strand. All PCGs started with ATG, except for *COX1* using GTG as the start codon. Eleven PCGs (*ND1*, *ND2*, *COX1*, *ATPase8*, *ATPase6*, *COX3, ND3*, *ND4L, ND5*, *ND6*, and *Cytb*) typically terminated with TAA or TAG as the stop codon; while *COX2* and *ND4* ended with incomplete termination codon T–. Twenty-two tRNA genes, 66–75 bp in length, displayed a typical clover-leaf secondary structure, except for *tRNA-Ser*^(^*^GCU^*^)^, which failed to form the dihydrouracil loop. The CR was 700 bp in length with high A + T content (67.42%), which was located between *tRNA-Pro* and *tRNA-Phe*.

To explore the taxonomic status of *E. rhadinum*, the phylogenetic tree was constructed with five species in Eleutheronema and 13 representative species in Perciforms based on their complete mitogenome. The results showed that all of the nodes were well-supported with high bootstrap values (Figure 1). This tree clearly demonstrated that *E. rhadinum* was closely related to *E. tetradactylum* of Eleutheronema. Together with *Pentanemus quinquarius*, *Polydactylus sextarius*, *Polynemus dubius*, and *Polynemus paradiseus*, they formed the monophyletic clade of Polynemidae. We expect that the present result will contribute to future taxonomic, systematic, and genetic studies of Polynemidae.

**Figure 1.**
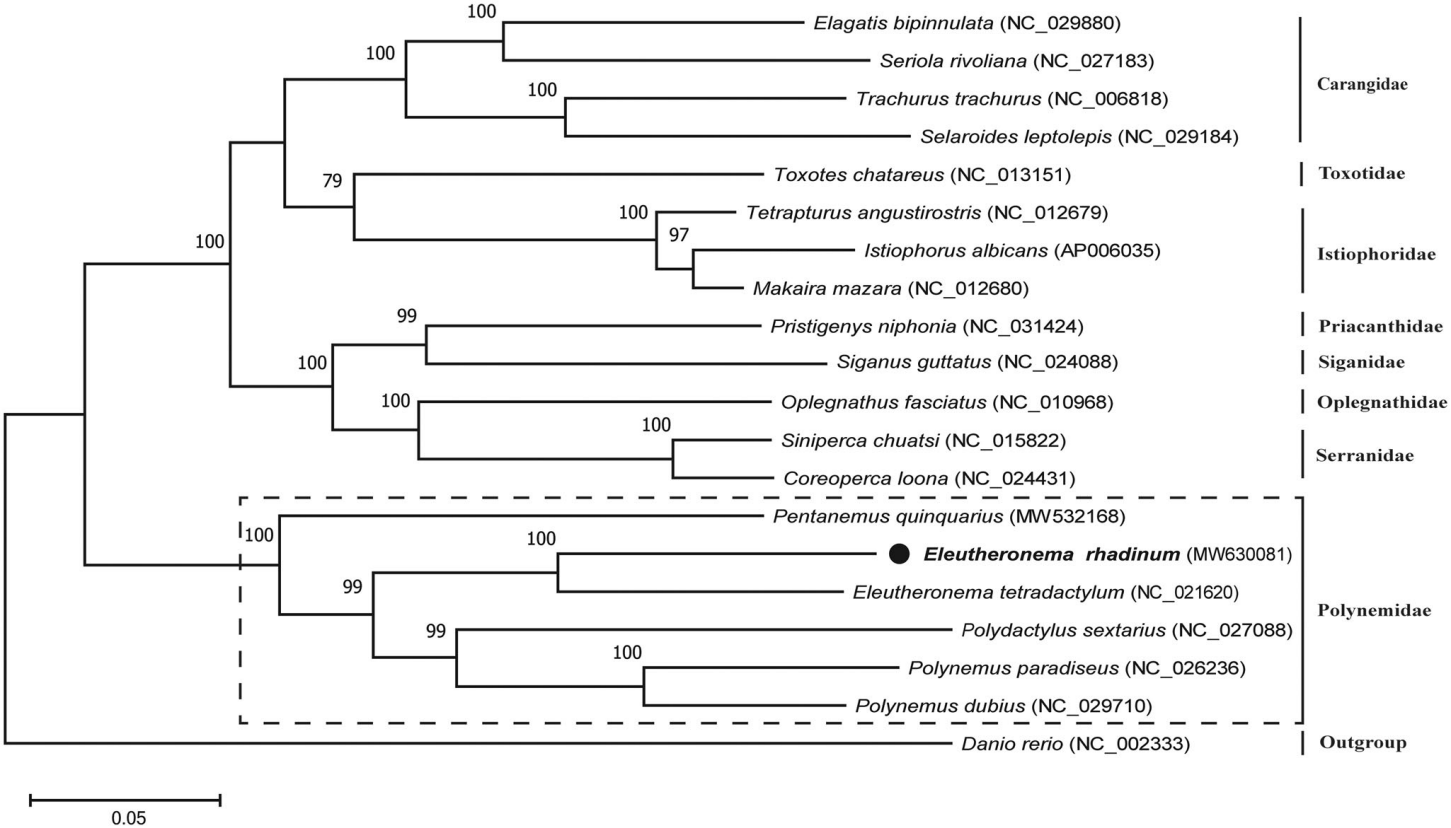
Phylogenetic analysis of *Eleutheronema rhadinum* and other fishes in Perciforms based on their complete mitochondrial genomes using maximum-likelihood (ML) method. The tree with the highest log likelihood (–159,095.88) is shown. Bootstrap support values (1000 replicates) are indicated at the nodes.

## Data Availability

The sequence data that support the findings of this study are openly available in GenBank of NCBI at https://www.ncbi.nlm.nih.gov under the accession number MW630081. The associated BioProject, SRA, and Bio-Sample numbers are PRJNA702826, SRR13741422, and SAMN17980855, respectively.
